# Quantum-Like Interdependence Theory Advances Autonomous Human–Machine Teams (A-HMTs)

**DOI:** 10.3390/e22111227

**Published:** 2020-10-28

**Authors:** William F. Lawless

**Affiliations:** 1 Departments of Mathematics & Psychology, Paine College, Augusta, GA 30901, USA; w.lawless@icloud.com; 2 Summer Fellow 2020, Naval Research Laboratory, Washington, DC 20375, USA

**Keywords:** interdependence, quantum-likeness, autonomous human–machine teams (A-HMTs), artificial intelligence (AI), roadmap, vulnerability

## Abstract

As humanity grapples with the concept of autonomy for human–machine teams (A-HMTs), unresolved is the necessity for the control of autonomy that instills trust. For non-autonomous systems in states with a high degree of certainty, rational approaches exist to solve, model or control stable interactions; e.g., game theory, scale-free network theory, multi-agent systems, drone swarms. As an example, guided by artificial intelligence (AI, including machine learning, ML) or by human operators, swarms of drones have made spectacular gains in applications too numerous to list (e.g., crop management; mapping, surveillance and fire-fighting systems; weapon systems). But under states of uncertainty or where conflict exists, rational models fail, exactly where interdependence theory thrives. Large, coupled physical or information systems can also experience synergism or dysergism from interdependence. Synergistically, the best human teams are not only highly interdependent, but they also exploit interdependence to reduce uncertainty, the focus of this work-in-progress and roadmap. We have long argued that interdependence is fundamental to human autonomy in teams. But for A-HMTs, no mathematics exists to build from rational theory or social science for their design nor safe or effective operation, a severe weakness. Compared to the rational and traditional social theory, we hope to advance interdependence theory first by mapping similarities between quantum theory and our prior findings; e.g., to maintain interdependence, we previously established that boundaries reduce dysergic effects to allow teams to function (akin to blocking interference to prevent quantum decoherence). Second, we extend our prior findings with case studies to predict with interdependence theory that as uncertainty increases in non-factorable situations for humans, the duality in two-sided beliefs serves debaters who explore alternatives with tradeoffs in the search for the best path going forward. Third, applied to autonomous teams, we conclude that a machine in an A-HMT must be able to express itself to its human teammates in causal language however imperfectly.

## 1. Introduction

Overview: In this article, we review why interdependence theory is fundamental to human teams and, generalizing from our case studies, why we expect it to be fundamental to autonomous human–machine teams (A-HMTs). We review why the effects from interdependence have made the science of human teams difficult to unravel, noting that without a mathematical metrics that incorporate these effects, it may be impossible to know whether A-HMTs are performing safely and effectively. As a step towards a mathematical metrics of interdependence, we review the quantum likeness of interdependence theory. Finally, by exploring the results based on the metrics we developed with interdependence theory, for future research, we review our discovery of “vulnerability” that motivates teams to avoid or exploit it, a new perspective of competition that not only accounts for mergers and spinoffs, but also maximum entropy production (MEP).

A situation. In late 2017, a “formidable” Russian base in Syria was attacked by a “highly sophisticated” swarm of drones; Russian soldiers were killed and aircraft destroyed, but Russia denied that any deaths of its soldiers had occurred from the surprise attack, and the U.S. denied any knowledge of the event [1]. The drones in this attack were thought to be controlled individually over a long distance by humans assisted by GPS guidance. More and more today, drones can rationally control themselves like a flock of birds following a leader, each drone monitoring the other, or with the lead drone overseen by humans (for a brief animation of Raytheon’s Coyote control system, see [2]). But for fully autonomous machines to be realized and applied to a human–machine system attempting to solve complex problems confronted by uncertainty [3], autonomous systems face many of the same issues of control human teams have experienced for eons, and maybe even new ones as both attempt to work together to exploit their complementary viewpoints, assets and skills [4].

Machines are not yet autonomous, but the capabilities of individual machines are improving dramatically. Compared to 2016, when three humans were needed to control one drone [5], the problem at that time was to find a way to invert the situation in control. By comparison, in warehouses today, humans are occasionally needed to bail out their robots, nonetheless, one human can now oversee eight machines equipped with both “eyes” and “brains,” an adaptation not used previously [6]. Even supermarkets have introduced several “autonomous cashiers” overseen by a single clerk.

The problem of autonomy, however, is unsolved by any rational mathematics [7], a foundational problem for autonomous machines made more challenging by the need to interact with humans as part of a team [4]. Yet, as we have claimed, no theory can solve this problem without a theory of interdependence [8,9]. To address autonomy, various approaches to interdependence will be briefly covered, including artificial intelligence (AI), systems engineering (SE), information fusion (IF), social systems theory and control theory. Interdependence is often paraphrased in social dynamics or in SE as a “whole greater than the sum of its parts” (e.g., [10,11]; respectively). Interdependence is fundamental to every social interaction ([4,12]); it transmits positive (synergism) and negative (dysergism) interference [9]; it occurs in large, coupled systems, such as the International Space Station (ISS; see [13]) where engineers designed ISS to dampen dysergistic effects. It can cause misinterpretations along the multi-step processes in complex information fusion systems; e.g., Llinas [14] issued a call “for action among the fusion, cognitive, decision-making, and computer-science communities to muster a cooperative initiative to examine and develop [the] … metrics involved in measuring and evaluating process interdependencies … [otherwise, the design of] modern decision support systems … will remain disconnected and suboptimal going forward.”

To advance the science of interdependence, we have explored how humans and machines build a context shared among teammates to solve the problems of autonomy [3]. We have proposed authorizing autonomous machines to override dysfunctional human operators to prevent tragic accidents (e.g., the Germanwings copilot who killed himself, 150 passengers and crew in 2015; in [15,16]). And we have studied innovation in the Middle East [8]. But here, we use case studies for a roadmap to autonomy by assuming that interdependence is quantum-like to explore uncertainty and non-factorability for future human–machine teams engaged in debate.

Traditional social data. Kenny and colleagues (p. 235, [17]) recommended to experimental social psychologists the removal of interdependence from experimental data to make their data i.i.d. (independent and identically distributed), somewhat akin to treating quantum effects at the atomic level as “pesky” [9]. Moreover, a leading social psychologist, Jones (p. 33, [12]), described interdependence as “bewildering.” Yet, Cooke [4] and her colleagues have conducted laboratory studies of interdependence in teams for over two decades. Interdependence may be too difficult to fully unravel here; but we can investigate its quantum likeness as a guide to advance the science of autonomy.

Interdisciplinary Approaches. The science of interdependence for autonomous human–machine teams may require contributions not only from AI (including machine learning, or ML), SE and IF, but also from other disciplines to find the approaches that allow humans and machines to best train and work together. For example, we borrowed from social science how context is interdependently constructed among teammates [3], and how trust is affected when humans and machines depend upon each other [18]; we borrowed from Human Factors research how human–machine teams should best perform work as a team [4]; and from AI, how human–machine teams need a bidirectional language of causal explanation ([19,20]; for today’s inability of causal explanations to be achieved by AI, see also p. 2, [21]). From other disciplines, we need to know why only equally competent opposing lawyers can provide justice [22], ethics to know the limits of morality, and sociology to guide the appropriate team behaviors across society and different cultures, the workplace, healthcare, and combat. We need to know from psychology the impact on humans when teaming with machines that can think faster than humans even in relatively mundane situations as with self-driving cars; the more complex but still traditional decision situations in combat (that is, “in-the-loop”; for example, with the Navy’s Ghost fleet; the Army’s self-driving combat convoy teams; the Marine Corps’ remote ordinance disposal teams); or the more intractable scenarios emerging with humans as observers of decisions (that is, “on-the-loop”), for example, with the Air Force’s attritable combat drones flying wing for an F-35 [23] as a “loyal wingman,” or alone on an autonomous combat mission flying across uncertain environments (i.e., the “Skyborg” project; in [24]).

Why quantum likeness? The past few years have been difficult for the social sciences which have suffered from the twin failures to replicate high-profile experiments (e.g., [25]) and to validate ballyhooed concepts. First, several scientific findings in recent years that have made headlines have turned out to be false and not reproducible [26]. NAS reported that many ordinary scientific findings published in peer-reviewed journals cannot be replicated, a severe problem from not following scientific procedures. Another reason offered by Lee Jussim, a leader in social science, is that the opposite of groupthink is an intellectual diversity which limits it [26]. He attributes groupthink to the social pressures that make us think and act alike. Per Jussim, the pursuit of scientific facts is plagued by uncertainty at every turn. He claims that intellectual diversity is crucial to resolving that uncertainty. But the findings of Cummings [27] contradict Jussim.

Consider that the National Science Foundation (NSF) welcomes new proposals from scientific teams for “bold, unconventional ideas incorporating creative interdisciplinary approaches,” (from https://www.nsf.gov/pubs/2014/nsf14106/nsf14106.jsp) a motivation for young scientists to add interdisciplinary members otherwise not needed to their team. Cummings ([27]; also in [28]), studied 500 science teams in NSF’s database to find that while the best science teams were fully interdependent, the worst performing scientific teams were interdisciplinary teams of scientists, a clear example of how a bureaucracy might inadvertently impede a team’s communications, its performance, and its ability to innovate.

Second, the lack of validity is a long-standing problem in social science. For example, twenty-five years ago, Bednar and Peterson [29] wrote that self-esteem was “generally considered to be a highly favorable personal attribute, the consequences of which are assumed to be as robust as they are desirable. Books and chapters on mental hygiene and personality development consistently portray self-esteem as one of the premier elements in the highest levels of human functioning… Its general importance to a full spectrum of effective human behaviors remains virtually uncontested. We are not aware of a single article in the psychological literature that has identified or discussed any undesirable consequences that are assumed to be a result of realistic and healthy levels of personal self-regard.”

That “single article” was published by Baumeister and colleagues [30] who found no evidence that self-esteem was associated with performance at work or academics. Today, society is focused on the pernicious effects of implicit racism, also found to be invalid [31], possibly why despite vast sums of money spent to counter it [32], the results have not been supportive [33]. However, one of Blanton’s coauthors criticizing the validity of implicit racism was Tetlock. In a new book, Tetlock and Gardiner [34] declared that forecasting is a skill that can be trained to be more rational. Gathering the best forecasters Tetlock and Gardiner could find and after honing their skills, these “superforecasters” predicted in 2016 that Brexit in the United Kingdom would not occur, and that Trump would not become president of the United States (e.g., for Brexit, see [35]).

Thus, we have come to believe that social science is too tied without question to rationality [8]. In social science, observations are assumed to provide independent evidence of actions; thus, an observation of an action supposedly captures that action. Simplicity itself, concepts in turn are designed to capture observations, whether applied in social theory, sophisticated computer models or games, but, as we have argued above [3], creating the dual crisis with replication and validity. As another example of this problem, a news report in *Science Magazine* [36] on an HIV prevention trial for the female mates of HIV males reported that:

The women reported using PrEP 90% of the time, and their unused returns seemed to validate that figure. But when the researchers later analyzed blood levels of drugs in the women, they found that no more than 30% had evidence of anti-HIV drugs in their body at any study visit. “There was a profound discordance between what they told us, what they brought back, and what we measured,” infectious disease specialist Jeanne Marrazzo said.

And as an example with AI, an impressive success of a recent simulation reported [37] that:

AI still has a long way to go before the Air Force pilots would be ready to hand over the stick to an artificial intelligence during combat, DARPA officials said during today’s live broadcast of the AlphaDogfight trials. But the three-day trials show that AI systems can credibly maneuver an aircraft in a simple, one-on-one combat scenario and shoot its forward guns in a classic, WWII-style dogfight.

However, for simulated air combat no matter how sophisticated a computer, the computer’s awareness of all combatant positions, speeds, and contexts precludes uncertainty; in comparison, for autonomous humans, convergence processes create uncertainty, which we address in closing.

Interdependence theory accounts for the failure of these concepts, predictions and simulations; surprisingly, interdependence theory suggests that some of these “invalid” concepts may instead be orthogonal, suggesting a fresh look (e.g., for implicit bias, see [38]). From Table 1 below, we can better understand the weaknesses of AI models based on traditional social science; e.g., invalid concepts such as self-esteem or implicit racism may simply be orthogonal, suffering from a measurement problem.

## 2. Materials and Methods

The design we use is to contrast theory, models and data. We begin by reviewing working definitions for interdependence; control; correlations; Kullback–Leibler divergence; maximum and least entropy production calculations (MEP and LEP, respectively); conjugate pairs; uncertainty; aggregation; and teams. For our Method, we compare case studies with Table 1, giving us our results.

Interdependence defined. We advance interdependence theory by mapping similarities between quantum theory and our prior findings; e.g., to maintain interdependence [9], we have established that boundaries are necessary for teams to reduce external dysergic interference (akin to preventing quantum decoherence); that redundancy interferes with team performance; that establishing context requires a consensus derived from the tension afforded by debate, competition or conflict; that AI-guided machines can rescue a transport (airplane, train, ship) operated by a dysfunctional human operator [15]); and that patent innovation is the byproduct of a highly-educated nation [8].

Cooke [4] and her colleagues have studied interdependence in the laboratory for at least two decades. Instead of the laboratory, we lay the groundwork with studies from the field for a mathematics of interdependence to at least bound some of its effects. Kenny et al. (p. 235, [17]) recommended removing interdependent effects in subjective data to make it i.i.d. But by adopting Kenny’s advice, traditional computational approaches to teams are unable to confront uncertainty. Instead, we recognize that interdependence between conjugate pairs makes uncertainty a part of every interaction, the ingredient missing in traditional models of the interaction [7]. That allows us to focus on the factors which impede interdependence (redundancy; [39,40]) or enhance it (the skills training for air combat, or the education for patent development; together, these two demonstrate orthogonal phenomena; in [8,9]).

We divide interdependence arbitrarily into three effects: Bistability; measurement problem; and non-factorabililty. In Table 1, this arbitrariness is evident by our repetition of the phenomena across the case studies.

Bistability: The spontaneity of two-sided views of reality are common in situations involving competition, innovation, and the uncertainty for which Kahneman [41] claimed individual humans handled poorly. In contrast, bistable views are unlikely to be expressed in situations associated with oppression. With a Kullback–Leibler model of divergence, we found that oil firms located in authoritarian governments had more redundant employees than those located in governments that permitted free markets [39], replicated for militaries [40]. We not only found that redundancy interfered with bistability, but also that it was associated with significantly more corruption under authoritarian governments; e.g., to illustrate, with Kullback–Leibler, we considered the effect of the oil shock in mid-March 2020 with a convenience sample of the five oil firms published in the figure on the front page of the *Wall Street Journal* [42] (i.e., Apache Oil; Cabot Oil; Chevron; Exxon Mobile; and Occidental Oil.) by contrasting their drop in stock market prices from year to date versus the one-day drop from the shock, normalized against their revenue per employee to capture redundancy; even in this small sample, we found a nearly perfect R-square of 0.9695, confirming that redundancy impedes the performance of a team before and during a shock.

Measurement Problem: Measuring an interdependent situation produces a single perspective, commonly modeled by a convergence process mathematically; in addition to a measurement, it could also represent the suppression of opposing viewpoints by an authoritarian leader [9]; or the minority control from seeking consensus that blocks a majority from acting [43].

Non-Factoribility. Many if not all social interactions are not factorable. Non-factorability leads to an uncertainty principle that is commonly addressed when debating in public, when solving intractable social problems, or when finding solutions under uncertainty by engaging in tradeoffs [8]; in contrast, conventional solutions assume that social data can be dis-aggregated and summed across agents.

**Table 1:** Some of the quantum-likenesses of interdependence.

Phenomenon	Interdependent Quantum-Likeness. Description	Case Study Numbers 1 to 6	Quantum Effects
Bistability	Two-sided views [8,9]; action-observation; two tribes with dissimilar opinions [44]; or debate (cf. in this paper).	Case 2: USS McCain Case 4: Uber Case 5. Tesla Case 6: DOE SRS-CAB	Photoelectric wave-quanta effect [45]. Wave-particle duality [46].
Measurement Problem	Measuring one aspect of interdependent orthogonal relations increases uncertainty in its interdependent co-factor; associated with accidents [15]; redundancy sheds light on the disruption of interdependence.	Case 1: USS Greeneville Case 2: USS McCain Case 4: Uber Case 5. Tesla Case 6. DOE Hanford CAB	Sketched by P. A. M. Dirac, re-formulated mathematically by von Neumann [47].
Non-factorability	Information subadditivity from maximum interdependence precludes the replication of a perfect team; e.g., divorcing couples or businesses go to court; armies fight; or, facing uncertainty, debaters test alternatives to weigh tradeoffs.	Case 3: USS Vincennes Case 6: DOE SRS-CAB	The no-cloning theorem indicates it is impossible to create an independent and identical copy of an unknown arbitrary quantum state (p. 77, [48]).

Control. Control has been a part of the human-centered design (HCD) that has been dominant in Systems Engineering for more than two decades [49]; HCD, also known as humans “in-the-loop,” is today the preferred process for Systems Engineering [50]. However, because a sequence of human-centered activities may not be independent, even for the simple acts entailed in driving a car, relatively recently, HCD was once considered harmful (e.g., [51]).

Similarly, but now looking towards the near future, autonomous systems are considered potentially harmful. For humans interacting with technology, a common refrain is that “we must always put people before machines, however complex or elegant that machine might be” [52]. On the positive side, since most accidents are caused by human error [15], autonomous machines may save more lives. But an editorial in the *New York Times* [53] warned that AI systems can be hacked, suffer data breaches, and lose control to adversaries. The Editors quoted the UN Secretary General, Antonio Guterres, that “machines with the power and discretion to take lives without human involvement… should be prohibited by international law.” The editorial recommended that “humans never completely surrender life and decision choices in combat to machines.”

Full autonomy raises the bar even higher. In the U.S., “Lethal autonomous weapon systems (LAWS) are a special class of weapon systems that use sensor suites and computer algorithms to independently identify a target and employ an onboard weapon system to engage and destroy the target without manual human control of the system” [54]. This concept of autonomy is known as human “on-the-loop” or “full autonomy.” Human “on-the-loop” observations of autonomous machines making self-directed decisions carry significant risks. But because the technology of autonomy is developing more rapidly than anticipated, these proposed rules for LAWS are already considered dated [55].

Correlations. A correlation measures the strength and direction of an association between two factors independent of each other (i.i.d.). A positive correlation means an increase in one factor is associated with an increase in another (e.g., height and weight), a negative correlation the opposite (e.g., elevation and air pressure). A correlation does not indicate causality; however, when conjugate factors in complementary pairs are measured, a zero correlation does not mean the absence of causality [8].

Kullback–Leibler divergence. This model of divergence, also known as relative entropy, measures statistically how the probability of a distribution, assumed to be a reference or baseline distribution, is different from a target distribution. The divergence of the target distribution from the baseline distribution is straight forward, except when working with subjective data (e.g., [36]). To address this, we ran our results through regressions to measure the similarity of two distributions [39,40].

Maximum Entropy (MEP) and Least Entropy Production (LEP), respectively. Compared to a smaller, less stable structure, a well-fitted structure that is also stable to external influences can produce maximum entropy in a non-equilibrium state, especially as its structure evolves [56]. We suspect that evolution occurs when an internal vulnerability arises during a competition along with sufficient free energy (negentropy) to transform the structure. Intelligence becomes necessary in a system to avoid an obstacle to reach its goal of achieving MEP [57]. In contrast, a stable structure requires that least entropy production (LEP) is necessary to achieve MEP [9].

*Conjugate pairs*: Two variables are conjugate if one is the Fourier transform of the other; e.g., if a Gaussian distribution has *σ* as its standard deviation, the standard deviation of its Fourier transform is 1/*σ*. Thus, measuring one variable conjugate with another increases uncertainty in the other; e.g., in signal detection theory, Cohen (p. 45, [58]) wrote that a “narrow waveform yields a wide spectrum, and a wide waveform yields a narrow spectrum and that both the time waveform and frequency spectrum cannot be made arbitrarily small simultaneously.”

Uncertainty. A conjugate pair of non-commuting quantum observables (i.e., Hilbert-space operators), enables mathematical predictions of physically observable quantities, such as position and momentum, subject to the uncertainty relations. Bohr [44] saw such relationships (here that of the exact position and the exact momentum measurement) as complementary, that is, as mutually exclusive and thus not applicable simultaneously, and yet equally necessary for a comprehensive account of the totality of the phenomena considered in quantum physics. Bohr generalized complementarity, in conceptual rather than mathematical terms (such as that of conjugate variables), beyond quantum physics. In particular, he argued human observation and action formed a tradeoff, such as an observer and an actor, or the political position of one tribe in comparison to another’s; to Bohr’s concept, we have added the interdependent skills that form the complementary relations that make a team successful (e.g., in a simple restaurant with a cook, a waiter and a cashier, when the humans in these roles are busy, they cannot capture the subjective information being observed by their complementary partners, forming a tradeoff in uncertainty that accounts for the failure of complementarity studies; reviewed in [9]). A generalization has led us to argue that resolving an uncertain context by a single agent is not possible, requiring instead a debate, competition or conflict between at least two agents to determine the uncertain context [3]; e.g., this problem often exists in relationships and businesses pursuing divorce proceedings against each other in a court of law where each side claims that its distribution of assets is best [59], creating a human tug of war that attempts to exploit the uncertainty tradeoff.

Aggregation. Reductionism from disaggregation is the usual approach to the data collected from teams or organizations. Based on Shannon’s [60] information theory, Conant [61] argued that interdependence in an organization should be minimized to maximize knowledge, meaning that joint information for Agents *A* and *B* should be:

(1) $$H_{A,B}\;\geq \;H_{A},H_{B}$$

But this approach produced a lack of validity for the expectation that managers of organizations should have deep knowledge about their organizations; instead, Bloom and his colleagues (p. 7, [62]) found that, “generally, subjects did not know how their management behavior compared against accepted practices or even with that of other firms in their sector, and answers to this question were not well correlated with either management practice score, or their own business performance. This situation applied in all regions, and did not change in better or more poorly managed firms (Exhibit 9).”

Bloom’s reductionism disaggregates data (Equation (1)). A different approach is needed to satisfy Baras [7]. We assert that in states of interdependence, aggregation becomes key to the effects of interdependence and understanding the mysteries of teams or organizations that exploit the power of interdependence.

Teams. Teams are inherently interdependent systems [28]. Teams and groups have been studied for decades, yet no mathematics of interdependence exists that can determine how the members of a team should fit together to best function as a team. Indeed, this claim about interdependence is similar to Einstein’s complaint to Bohr [63], “our ordinary intuition about physical systems is that if we know everything about a system, that is, everything that can in principle be known, then we know everything about its parts. … But Einstein explained to Bohr—in quantum mechanics, one can know everything about a system and nothing about its individual parts.”

In response to the Einstein–Podolsky–Rosen [64] paper, Schrodinger coined the term ‘entanglement’ to describe a peculiar connection between quantum pairs (p. 555, [65]), “attention has recently been called to the obvious but very disconcerting fact that even though we restrict the disentangling measurements to *one* system, the representative obtained for the *other* system is by no means independent of the particular choice of observations which we select for that purpose… Another way of expressing the peculiar situation is: the best possible knowledge of a *whole* does not necessarily include the best possible knowledge of all its *parts*, even though they may be entirely separate and therefore virtually capable of being ‘best possibly known,’ i.e., of possessing, each of them, a representative of its own. The lack of knowledge is by no means due to the interaction being insufficiently known—at least not in the way that it could possibly be known more completely—it is due to the interaction itself.”

Based on Einstein’s and Schrodinger’s claims, we use Equation (2) to reflect the interdependence that makes a “whole greater than the sum of its parts” (also, [10,11], respectively):

(2) $$S_{A,B}\;\leq \;S_{A}\;+\;S_{B}$$

Later in his book, *What is life*?, Schrodinger [66], extended his quantum ideas to biology, beginning by asserting that life is lived at a “fairly low entropy level,” but in the epilogue, he argues about identifying (in one’s mind) the human and the devine, when he writes that, “those true lovers who, as they look into each other’s eyes, become aware that their thought and their joy are numerically one—not merely similar or identical.”

We suspect that Schrodinger was not being a romantic (e.g., [67]), but that he was extending entanglement to interdependent states. To capture this idea, we propose that, in the limit,

(3) $$lim_{dof\to 1}log\left(dof\right)=\;0$$

Equation (3) helps us to understand why, despite decades of study, there is little to show to unravel the power of teams. Mindful of this long-lived failure to appreciate interdependence in teams or organizations, we assert that it cannot be predicted which of two parts, *B* or *C*, best go with part *A*, making replication similar to the no-cloning theory of Wooters and Zarek (p. 77, [48]):

(4) $$human\;-\;teammate_{A}\;+\;human\;-\;teammate_{B?-or-C?}$$

However, instead, if the freedom exists to pursue trial and error, then we can generalize Schrodinger:

(5) As a team’s structural fitness reduces its LEP, its capability increases to reach MEP

Equation (5) is supported by the successful mergers that have arisen from the consolidation of two firms, two markets, two churches, a marriage, the formation of a new team, all characterized by a reduction in structural LEP that, if successful, maximizes MEP to improve a team’s competitiveness [40].

## 3. Results

For our results, briefly, we review six case studies from the field; afterwards, we compare those results with Table 1. First, a shipwreck caused by the USS Greeneville in 2001; then a collision caused by a U.S. Navy destroyer; the shoot down of an airbus; a fatality caused by a self-driving car; Tesla’s near miss of meeting its quarterly quota; and the U.S. Department of Energy’s (DOE) decision to restart closing its high-level radioactive waste (HLW) tanks.

1. An order given by the Commanding Officer of the USS Greenville resulted in the deaths of Japanese tourists. With distinguished visitors aboard the submarine who had just witnessed its dive to depth under the sea, the commanding officer ordered the crew of the submarine to make a rapid ascent that, once at the surface, struck and sank the Ehime Maru, a Japanese tour boat, killing nine of its passengers [68].
2. In 21 August 2017, the US Navy destroyer John S McCain collided with the tanker Alnic MC. The McCain was overtaking the Alnic in the westbound lane of the congested Singapore Strait when the destroyer’s watch-standers perceived a loss of steering. As its crew sought to regain control, the destroyer turned to port and collided with the tanker. In the collision, 10 McCain sailors died, 48 were injured, and the destroyer sustained over $100 million in damage; no one was injured aboard the Alnic MC and it sustained only minor damage. NTSB [69] attributed the accident to the watchstander’s unwitting transference of steering control from the McCain’s helm to its lee helm, causing a loss of situational awareness on the destroyer’s bridge, coupled with the lack of automatic identification of the McCain’s presence to other ships in the Singapore Strait.
3. During seven critical minutes after Iranian Flight 655 was airborne, the Captain of the USS Vincennes and his watch team were acting on several events (from [70]; also [71]). The Vincennes, a guided missile cruiser, was engaged in a high-speed surface battle with at least two groups of Iranian small boats, each with the ability to make a suicide run against it or its two sister ships. One of the cruiser’s helicopters had come under attack from the Iranian small boats. She was tracking an Iranian P-3 military aircraft 60 nautical miles to its northwest that was providing information to Iranian attack aircraft. The Captain had been given tactical command of the two nearby U.S. ships and he was about to assume command of U.S. combat aircraft approaching from outside the Persian Gulf. The Vincennes had a fouled gun mount that required extensive maneuvering to keep its remaining gun aimed at multiple threats, at one point making a full rudder turn at 30 knots, causing the ship to heel sharply, adding tension to the situation. Then, an unidentified Iranian commercial airbus took off from a joint military/civilian airport headed directly towards the Vincennes, the same airfield from which Iran had recently launched multiple fighter attacks on U.S. naval forces. The airbus was 27 min behind any scheduled commercial airline departure from this airport. It was flying in a known commercial air corridor, but it was off the usual centerline some 3 or 4 miles. Initially, the Vincennes identified the unidentified contact as a combat fighter jet; it was increasing speed; and it did not respond to repeated challenges from the cruiser over military and international emergency distress frequencies, all combining to offer a tradeoff: On the one hand, the threatening contact was closing about 5–6 miles a minute; on the other, a decision to defend the ship and crew had to be made before the unknown contact got closer than 10 miles. But at a range of between 15 and 12 miles, the Vincennes’ Tactical Information Officer (TIO) reported that the altitude of the unidentified contact was decreasing, leading to the decision to down the Iranian airbus. Later investigation did not support the TIO’s claim that the contact’s altitude was decreasing.
4. In 2018, the sensors of a self-driving Uber car at night detected an obstacle 6 s ahead of it in the road on which it was traveling. The Uber car selected its brakes 1.3 s early, but the brakes had been disabled by engineers to help the car ride better. The car’s human operator saw the pedestrian 1 s early and hit the brakes 1 s after striking the pedestrian, killing the pedestrian [72,73].
5. In 2017–18, BMW’s human–robot teams were functioning synergistically, motivating BMW to add new employees and machines [74]. In comparison, Tesla’s all-robot factory struggled to meet its quota of 5000 cars per quarter [75], a dysergic system effect that Tesla’s human operators and robots were unable to solve by sharing their views of the problem with each other on the fly. To make its quota, Tesla ripped out and replaced many of its robots with humans.
6. Before 1983, the U.S. Department of Energy (DOE) used the minority control of its scientists to prevent criticism of its use of cardboard boxes to dispose of 95% of its solid radioactive military wastes [43]. After its practices were publicized, DOE was publicly embarrassed; in 1993, DOE created citizen advisory boards to help it to recover its lost good will. With DOE guidance, the DOE Hanford Citizens Advisory Board (CAB) in Washington State chose to use consensus-seeking decisions (another form of minority control) that promoted hostility rather than collegiality [76] and blocked DOE’s Hanford facility from closing its high-level radioactive waste (HLW) tanks. DOE Hanford recently began its first HLW tank closure [77]. In contrast to the impediments imposed by the consensus-seeking rules on the Hanford Board, (For more on the pitfalls of consensus-seeking, see the European Union conclusion that (p. 29, [78]): “The requirement for consensus in the European Council often holds policy-making hostage to national interests in areas which Council could and should decide by a qualified majority.” Also, an experiment performed in consensus-minded Japan found that compared to consensus-seeking rules, majority rules offered a richer set of considerations for the siting of a HLW repository in Japan [79]) majority-rules used by DOE’s Savannah River Site’s Citizens Advisory Board (SRS-CAB) in South Carolina accelerated cleanup at SRS; e.g., with the Board’s support, SRS closed the first two HLW tanks under regulatory oversight in the world in 1997 and several since. Further, its majority rules have promoted a sense of collegiality, satisfaction and accomplishment [76].

## 4. Discussion

First, we review the six cases above from the perspective of interdependence theory (see Table 1).

USS Greenville. In the case of the USS Greenville, the submarine Commanding Officer’s command given to his crew in front of VIP visitors prevented the crew’s feedback of alternatives inherent in bistability when facing uncertainty. Proceeding like a measurement problem, this case illustrates that a commander of a ship has the authority to make a decision that overrides alternative (bistable) perspectives. Most accidents are caused by human error [15], exemplified by the order given by the Greeneville’s commander. A machine, however, can be trained to prevent human operators from enacting a decision that threatens human life, similar to an airplane taking over from a human fighter pilot suffering from a loss of consciousness due to excessive g-forces, known as G-LOC [16].

USS McCain. With an effect similar to the measurement problem fostered by minority control, the second case study was about the loss of context [3,69] from the lack of attention among the members of a team who should have been multitasking together with their bistable attention divided between the destroyer’s navigation and its control; instead, as the McCain’s crew struggled to regain control of their ship in congested waters, their multitasking collapsed into single tasking as they ignored the ship’s navigation.

USS Vincennes. In the case of the USS Vincennes, its captain and crew may not have been aware of their heightened arousal from the dramatic events unfolding before them. Facing uncertainty, the Captain and his crew did engage in tradeoffs reflected by non-factorability; however, interdependence theory predicts that the best decisions facing uncertainty are made by a team at its ground state, the worst decisions at elevated (emotional) states [40].

Uber. AI’s Machine Learning (ML) program learned patterns sufficiently well enough to drive an Uber car. If we consider the human and machine as an interdependent system, by failing to share its bistable perspective by alerting its human operator seconds earlier than the human operator became aware of the pedestrian’s presence in the roadway, the Uber self-driving car became a poor team player [3]. Having the same effect as a measurement problem, the latter is an example of dysergy, the opposite of synergy, where team members do not support each other.

Tesla. It robots unable to tell its human operators what they could not see, again, having the same effect as a measurement problem, subsequent analysis discovered that Tesla’s human operators could not see that its machines were dysfunctional when placed in the unusual orientations they needed to assume on the assembly line, a problem solved with improved robotic vision [80]. Later, its robots evolved, synergy increased Tesla’s production beyond its original quota to over 80,000 in 2018′s Quarter 3 [81], continued in 2019 [82].

DOE. DOE’s embarrassment regarding its use of cardboard boxes to dispose of its solid radioactive wastes contradicts the claims by Sunstein and Vermeule [83] that government administrators act morally to protect the public’s interests. Based on DOE’s own use of minority control, DOE promoted consensus-seeking rules and was dismayed when DOE’s SRS-CAB not only rejected those rules in favor of majority rules, but also became successful with them, instilling a feeling of comity among SRS-CAB’s members compared to the DOE Hanford’s CAB [76].

Consensus-seeking rules, where anyone can block a decision, allow a minority to control decisions [43], preventing action when facing uncertainty. The problems with minority control can be generalized; e.g., innovation in China. In China [84], “Small private-sector firms often only have access to capital through expensive shadow banking channels, and risk that some better connected, state backed firm will make off with their designs—with little recourse.” General M. Hayden, the former Central Intelligence Agency (CIA) and National Security Administration (NSA) chief, regarding the innovation that has eluded China, told his Chinese counterparts [85]: “you can’t get your game to the next level by just stealing our stuff. You’re going to have to innovate.”

Consider that the R&D expenditures by China are second in the world to the U.S. [86]. But China’s state directed finance, its weak intellectual property protections and its rampant corruption have impeded its successful innovation.

In contrast to the consensus-seeking rules by the DOE Hanford CAB ([43]; or the European Union; in [78]), majority rules promote debate between adversaries who commonly stake out orthogonal positions as they work through tradeoffs. One of the advantages with majority rules is that the debates encourage neutrals to process the information to be weighed, thereby making neutrals those who judge the outcome of a debate.

**Figure 1:**
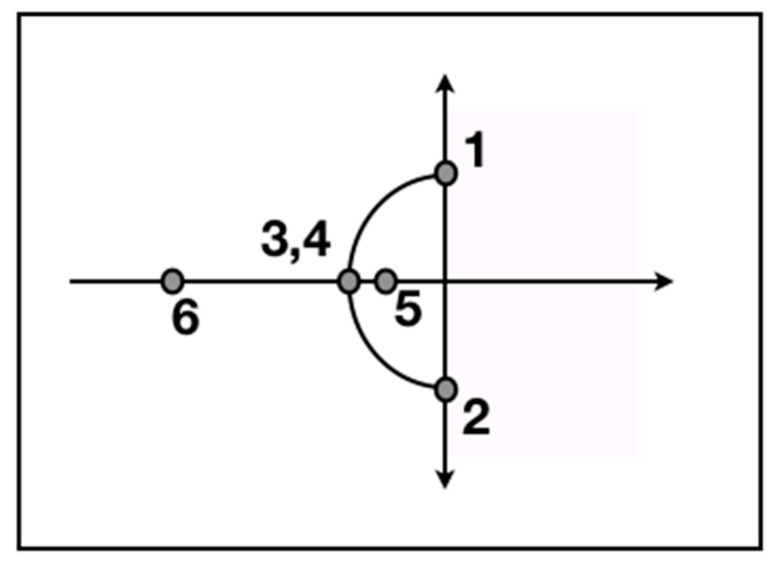
(From [9]). Points 1 and 2 on the *y*-axis represent beliefs posed on the imaginary axis [87] as a debate ensues over the choices for action; points 3 and 4 reflect a compromise choice for action on the *x*-axis (physical reality); points 5 and 6 represent resistance to the debaters that their audience has decided on the action it wants to be executed, with point 6 reflecting the strongest social feedback.

The power of majority rule by the SRS-CAB became evident in late 2011. Starting in 2005, based on a new law that allowed DOE to resume the closures of its HLW tanks, the new law also required NRC’s approval before any closures by DOE. After arguing with NRC for almost 7 years (points 1 and 2 in Figure 1 below), DOE’s inability to reach a compromise with NRC on the restart of closing DOE’s HLW tanks in 2011 led to the public stepping in. By doing so, the SRS-CAB brought fierce pressure against DOE and NRC to take action, modeled as resistance (point 6 in Figure 1).

We model the potential to change when free energy, α, is available, with the change of α per unit of entropy (dα/ds), by modeling as if it were in a circuit:

(6) $$d\alpha /dt\;=\;d\alpha /ds*ds/dt\;=\;\tau_{A}\omega_{A}\;=\;\tau_{B}\omega_{A}$$

Equation (6) suggests the possibility of a metric that reflects a decision advantage (DA), based on the speed of oscillation from the power of a debate, as one side seeks to gain an advantage over another:

(7) $$\mathrm{DA}\;=\;\tau_{output}/\tau_{input}$$

In the future, we propose a revision of Bayes theorem that incorporates the exploration of advantage in a debate, measured by the entropy produced to achieve MEP, and simulated in a Markov model.

## 5. Discussion: New Theory

The “Machiavellian intelligence” (MI) hypothesis is based on competitive social interactions as the most important factor driving evolution based on strategies of achieving social success at all costs, for example, with deception, manipulation, alliance formation, exploitation of the expertise of others, and the skills to learn and use them [88]. Once the mental tools for inventing, learning, and using these strategies occur, MI can address a variety of challenges such as political, environmental, ecological, technological, linguistic, and so on. Next, we propose that identifying, seeking, or creating vulnerability in opposing forces is a key tool.

We begin with Justice Ginsburg [89]. From her unanimous ruling, we know the value of competition among the courts and armies of legal and political experts as an important case winds it way to the Supreme Court when she argued that the process not be short-circuited because the intellectual battles provide an “informed assessment of competing interests…”

But how do we know who has won in a competition? Scores can be kept; patents can be issued; court cases can be won. Still, we are left with a question about what it means to compete or even how to win before a win is realized. If the goal of a team is to win, how does the process proceed, how does it know it is winning, and how generalizable is it? If the goal of a team is to achieve maximum entropy production (MEP; [9]), in a competition where one team competes against equal others, thereby increasing uncertainty, with a goal to win, a sub-goal in a competition between two evenly matched teams might be to find a vulnerability in an opposing team’s defenses indicated by a decrease in the weaker team’s MEP as it reduces productivity to shore up the vulnerability in its structure, presenting a clear signal of damage done.

If a vulnerability is exposed, it should be characterized by the reduction in the entropy generation of an opponent’s work output (MEP), along with a parallel increase in its structural entropy production (SEP) as the opponent engages in a tradeoff when it attempts to shore up its structural defenses; e.g., [90]: “China sees its dominance in strategic rare-earth minerals as leverage that can be used… in trade disputes with the U.S…. [which has forced the U.S.] Defense Department [to start] a new effort to bolster the U.S. supply chain, announcing grants this spring to help develop a processing facility at the only U.S. rare-earths mine, Mountain Pass in California, and at a new plant proposed for Texas.”

The richness of interdependence theory suggests a generalization by telling us how one aspect of competition, namely seeking vulnerability in an opponent, is enacted. It reintroduces rationality into a practical weighing of tradeoffs (e.g., the weights by politicians in their value judgements of cost-benefit analyses, in [91]; weights assigned to the acceptance of potential college students against whether they will accept, in [92]; the weights by Gilead whether to partner or merge to “diversify into cancer” as an offset to the declining sales of its other drugs; in [93]). And unlike social “vulnerability theory” with its focus on dependent individuals [94], the interdependence entailed by debate opens a new window into an opposing team’s awareness of its vulnerability that itself can be generalized to research on a wide range of human activities. To illustrate with a series of examples, the interdependent actions taken by humans to enact defensive organizational mergers (e.g., Accenture’s attempt to keep its lead in AI consulting; in [95]) (e.g., while Accenture claims the lead in applied AI consulting, it continues to acquire companies to strengthen its lead in data analytics services [95].) and spinoffs (e.g., Dell’s fear that its stock is undervalued; in [96]); (e.g., as an example of dysergy, Dell is considering spinning off its large stake in VMWare because the valuation of this stake by the stock market indicates that its data-storage business has virtually no worth [96].) to build military deterrence (e.g., [97], recommendation to counter Russia’s new Kanyon missile); (e.g., from Portzer [97], Russia’s Kanyon is a nuclear-powered unmanned, underwater vehicle that can travel thousands of nautical miles at 100 knots and a depth of 1000 m with conventional or nuclear warheads of about two megatons. Russia designed it as a strategic weapon to take out ports and coastal cities. The Nuclear Posture Review [98] describes the Kanyon as a “new intercontinental, nuclear-armed, nuclear-powered, undersea autonomous torpedo.”) to deploy robots that reduce the vulnerability of firms to covid-19 (e.g., [99,100]); (To safeguard firms and their revenue from the disruption caused by the covid-19 virus to the flow of goods around the world, especially products from China, Katzeff [99] described how firms have taken advantage of distribution networks and warehouses with robots and smart machines in a system wired with floors, walls, and ceilings that, coupled with Bar codes, sensors and radio ID tags on merchandise, talk to conveyor belts, carts, shelves, fork lifts and self-propelled automated guided vehicles (AGVs). Human workers wear smart eyeglasses to know where to place packages in AGVs. Orchestrated by integrated warehouse management software (WMS), robots perform monotonous, unsafe, and stressful tasks while the humans decide how and where to put more robots to work) to uncover deception (e.g., after the exposure of fraud, respectively, the recent collapse of both Wirecard, in [101]; and Luckin Coffee, in [102]); (Wirecard, the global electronic-payments giant, briefly Germany’s most valuable bank, unraveled with the disclosure that it used fictitious revenue to inflate its sales [101]. Luckin Coffee “revealed that… [some of] its 2019 sales were fabricated by some employees… Nasdaq last month determined that Luckin should be delisted because of “public interest concerns” arising from the company’s disclosure of fabricated sales and the company’s “past failure to publicly disclose material information,” the company said [102].) to use deception to defeat a superior force (e.g., in 480 B.C., the Greeks led by Themistocles used a hoax to defeat the superior Persian Fleet in the naval battle of Salamis; see p. 248, [103]); and to better understand a factory (e.g., the interdependence arising from a division of labor in a pin factory in an attempt to establish a process that removes vulnerabilities; [104]) or the interdependent attack in a stock market by short sellers (e.g., [105]). Included in this list should be the information arms race between plants and herbivores [106]. In the latter, Zu and colleagues [106] offer an example of interdependence among plants and herbivores engaged in a race for the “functioning and persistence of systems where individuals send and receive information in the form of signals to which other individuals react and, in turn, affect the behavior of other participants in these systems.”

To belabor the point, by suppressing contrary (bistable) views, the interdependently mis-perceived lack of vulnerability common in a dominant firm can lead it to make poor management decisions, as in the case with Boeing’s development of its 737 Max (e.g., from [107]): “dominant firms not only concentrate power but become the single sources for vital products. In 1997, Boeing and McDonnell Douglass merged, combining nearly all domestic civilian aerospace capacity in one company. While Boeing (as the new entity continued to be called) still faced some competition from Airbus, its market power largely insulated it from the consequences of poor management. The deadly crashes over the past year of two Boeing 737 Max passenger airlines have now begun to reveal the extent of the company’s failings.”

Alternatively, the software for autonomy itself can become a source of vulnerability; e.g., in vehicles bound for space, humans may be needed to override software vulnerabilities, like in the case of Boeing’s capsule that missed the International Space Station, its intended target (e.g., from [108]): “Boeing’s Starliner capsule (the other vehicle NASA is counting on to send American astronauts into space) failed to make it to the ISS because of a glitch in its internal timer. A human pilot could have overridden the glitch that ended up burning Starliner’s thrusters prematurely. NASA administrator Jim Bridenstine remarked soon after Starliner’s problems arose: “Had we had an astronaut on board, we very well may be at the International Space Station right now.”

But in the same news article by Patel [108], the SpaceX Crew’s Dragon capsule safely docked autonomously at the ISS, suggesting that the role of humans may be interdependently vulnerable to autonomous systems in the future of spaceflight: “The SpaceX astronauts may still be involved in decision-making at critical junctures, but much of that function has moved out of their hands. Does this matter? Software has never played a more critical role in spaceflight. It has made it safer and more efficient, allowing a spacecraft to automatically adjust to changing conditions. According to Darrel Raines, a NASA engineer leading software development for the Orion deep space capsule, autonomy is particularly key for areas of “critical response time”—like the ascent of a rocket after liftoff, when a problem might require initiating an abort sequence in just a matter of seconds. Or in instances where the crew might be incapacitated for some reason. And increased autonomy is practically essential to making some forms of spaceflight even work.”

Using game theory and the assumption that behavior aggregates directly, Schelling [109] concluded that a nation can strengthen its position by becoming more vulnerable, but then changed his conclusion in his Nobel prize lecture [110]. In battle, Hart (in [111]) concluded similarly that, the adversary’s strength should be circumvented in favour of indirect blows against his more vulnerable points. By choosing and exploiting the line or course of least resistance, the adversary’s equilibrium could be offset, causing dislocation.

Businesses, too, are well aware of exploiting vulnerabilities, a driving force for mergers [112], In the midst of the pandemic, Nvidia Corp. Chief Executive Jensen Huang engineered his company’s biggest game changer… The proposed acquisition of Arm Holdings… in the chip industry’s biggest deal ever, would transform Nvidia into a force across the most important arenas of computing, from servers and PCs to consumer electronics to smartphones, making it one of the most formidable threats Intel Corp. has ever faced.

And in philosophy, which has long used debate as a tool, Cicero argued that, even though truth may remain elusive, only with debate can the likelihood of truth be enhanced by weighing both sides of a debate [113]. Supporting the DOE’s SRS-CAB’s use of majority rules, Aristotle argued for the value of an audience (neutrals) to make a judgment [114]. Wittgenstein argued in favor of the principle of verification in logical positivism to connect language and the world [115], but then against a language’s connections to reality with his discussion of the multiple interpretations of “meaning” in his *Philosophical Investigations* (p. 218, [116]): “Meaning it is not a process which accompanies a word. For no process could have the consequences of meaning.”

Wittgenstein would not have been surprised by today’s struggle to interpret quantum theory (e.g., see p. 4, [117]: no consensus exists today on the interpretation of quantum mechanics). Weinberg [118] begins his search for a rational solution in his “*Trouble with Quantum Mechanics*”: “It is a bad sign that those physicists today who are most comfortable with quantum mechanics do not agree with one another about what it all means.”

However, there is another way to view these quandaries. If interdependence signifies life, and if bistability is the signature of intelligent life, then long-running debates promote innovation.

This inability to reach a definitive “meaning” returns us to what the “control” of A-HMTs may mean in practice. With the goal of rational control, by rejecting the cognitive model, physical network scientists [119] and game theorists [120] dramatically improve the predictability of behavior in situations where beliefs are suppressed, in low-risk environments, or for contexts where economic beliefs exist in highly certain environments. But the predictability by these rational models fails in the presence of uncertainty or conflict ([121]; for a list of pitfalls, see [122]), leading Kahneman ([123]; also, [124]) to label the average individual human’s choices as irrational. But conflict and uncertainty are exactly where interdependence theory thrives; e.g., the bistable effects (e.g., two-sided narratives composed of independent or orthogonal views) in debating the possible tradeoffs to choose a path going forward [9]. Facing uncertainty, debate exploits the orthogonal views of reality that exist to explore interdependently the tradeoffs that test, or search, for the best paths moving forward. Generalizing from the case studies, reducing uncertainty for a system necessitates that human and machine teammates must both be able to explain to each other however imperfectly their past actions and future plans in causal terms.

The goal for MEP seemingly contradicts those set for efficiency (e.g., in favor of efficiency, see the economist Coase [125]; human factors scientists [126]; social network theorists, Centola and Macy [127]; for those against efficiency, see military leader McCrystal et al. [128]; in business, see Christensen in [129]). To help us sort through this debate, Gold and Evans [130] asked and answered in the title of their article where they wrote, “Why did Covid overwhelm hospitals? A Yearslong Drive for Efficiency.” Buried in their article, however, they conclude that: “Less financially strong hospitals, which tend to be public or rural, were more vulnerable.”

That quote is what Doyle and his team found using a rigorous application of control theory to biology [131]. They reported that not only are engineering and evolution constrained by trade-offs between efficiency and robustness, but also that when applied to glycolysis, its interplay of feedback control and autocatalysis of network products generate universal and fundamental trade-offs between robustness and efficiency with oscillations that generalize to the robust efficiency of any autocatalytic network. We take Doyle’s results to mean that when a team’s structure, like the best run hospitals, operates efficiently, it not only has the energy available to reach MEP, but it is sufficiently robustly adaptable to uncertainty.

Human teams cannot be controlled in Doyle’s technical sense applied to the direct control of a swarm of drones to follow a drone leader, even if the leader is controlled by a human operator. Autonomous human team members function in specialized roles that require independent skills while working together with teammates interdependently [4]. While we agree with Doyle over the control of a team’s structure, we conclude that the direct control of fully autonomous human–machine teams, with each teammate performing its own unique (orthogonal) role and with a system of autonomous machines and humans designed to operate in uncertain contexts or under conflict, is unlikely and must instead be governance (e.g., [132]).

## 6. Conclusions

Until now, rational theory has been unable to model teams. We know that the best teams are highly interdependent [27], leading us to conclude that a mathematics of interdependence is necessary to model, build and operate teams, including A-HMTs. In that interdependence is quantum-like has given us a roadmap to the future development of interdependence theory and its mathematics.

Our first successful prediction with interdependence theory, that redundant teammates reduce a team’s productivity [39], followed from the two findings by Cummings [27] that the best teams were highly interdependent and that interdisciplinary science teams were the least productive teams.

But there is more. Interdependence theory may help social scientists to salvage concepts that they have been unable to validate. It may help AI computer simulations to become more real in their effects. And in this study, the richness of interdependence theory allowed us to gain insights into an opponent’s vulnerability that can be generalized for research across a wide range of seemingly unrelated human activities, such as organizational mergers; military deterrence; philosophical, religious and legal theory; politics; deception; market theory; and even to the information arms race between plants and herbivores.

In that no single human or machine agent can determine social context alone [3], resolving uncertain contexts requires a theory of interdependence to build and operate safely and ethically autonomous human–machine systems [133]. A team’s intelligence has been located in the interdependent interactions among its teammates [4]. By redefining a system to include the drivers of a debate, we extended these findings to the open-ended debates that explore tradeoffs seeking to maximize a system’s production of entropy (MEP) in competitive but highly uncertain environments.

The rational model fails when faced by conflict and uncertainty, where interdependence theory thrives. Unlike the rational theory of behavior, interdependence theory has passed every test posed to it, and it has led to new discoveries and insights.

For future research, the entropy production for novices should be more than for experts on a specific task. But that misleads. Compared to a novice, an expert addressing a previously intractable problem will apply more entropy focused on solving the previously unsolved problem, achieving MEP. Comparing novice and expert teams, the former will waste copious amounts of unfocused entropy; however, the latter will waste little entropy of their team’s structure but apply prodigious amounts of entropy, MEP, searching for an innovative solution to a target problem. Entropy then is a two-step problem: team structure and task; i.e., the perfect team minimizes its team structure’s entropy production to maximize its team’s performance.

As part of this roadmap, we are mindful of Machiavellian intelligence and the findings that a perfect team is more productive than the sum of the individuals who compose the team [27], but why? This problem has been studied by generations of scientists, producing lots of experimental data but no equations (e.g., [28]). The members inside of a team performing teamwork and outside of a team collectively performing the same work should generate entropy equally. But in a perfect team, some of that entropy inside of a team may be offset by another teammate’s entropy. Then, also future work, with a perfect team, what features at the individual level have an effect at the team level [4]?

Finally, we close by addressing the problem with the concept of computational convergence. Mathematically, convergence strengthens a story; e.g., dynamical systems are stable when small perturbations converge to stable trajectories; or converging solutions of differential equations may indicate equilibria. But autonomous humans have long accepted that convergence in non-factorable situations only occurs by making a tradeoff that automatically involves uncertainty. From the poem by Frost [134],

Two roads diverged in a yellow wood,
And sorry I could not travel both
And be one traveler, long I stood
And looked down one as far as I could
To where it bent in the undergrowth; …
I took the one less traveled by,
And that has made all the difference.

Convergence processes may lead to solutions with differential equations or system dynamics for dynamic but non-autonomous systems, where the separation of observation and object are safely assumed [44]; e.g., games of competition; large coupled systems; subjective surveys of multiple tribes. Autonomy, however, introduces the quantum-likeness of interdependence into every social situation, including where choice, deception and uncertainty operate and when autonomous systems are engaged in the changes from the feedback that makes systems dynamic, not only the system for which a solution is being sought, but also its autonomous opposition, the very wisdom of Ginsburg’s “assessment of competing alternatives,” [89], Frost’s *The road not taken*, [134], Clausewitz’s *On War* [135], and Bohr’s complementarity between two tribes [44]. Hidden by uncertainty [3], we may not realize what we are requesting when we want human–machine teams to become autonomous (A-HMTs), but, at a minimum, we want them to keep humans safe, to help humans to avoid the accidents illustrated in our Case Studies, and to assist humans to achieve success by addressing vulnerability; for these goals, we want our machines to apply the fullest capacity of their assessments with AI to intelligently monitor uncertainty, reduce accidents, avoid tragedies, and win competitions interdependently.
